# *In vitro* acellular dissolution of mineral fibres: A comparative study

**DOI:** 10.1038/s41598-018-25531-4

**Published:** 2018-05-04

**Authors:** Alessandro F. Gualtieri, Simone Pollastri, Nicola Bursi Gandolfi, Magdalena Lassinantti Gualtieri

**Affiliations:** 1 Department of Chemical and Geological Sciences, The University of Modena and Reggio Emilia, Modena, Italy; 2 Department of Engineering “Enzo Ferrari”, The University of Modena and Reggio Emilia, Modena, Italy

## Abstract

The study of the mechanisms by which mineral fibres promote adverse effects in both animals and humans is a hot topic of multidisciplinary research with many aspects that still need to be elucidated. Besides length and diameter, a key parameter that determines the toxicity/pathogenicity of a fibre is biopersistence, one component of which is biodurability. In this paper, biodurability of mineral fibres of social and economic importance (chrysotile, amphibole asbestos and fibrous erionite) has been determined for the first time in a systematic comparative way from *in vitro* acellular dissolution experiments. Dissolution was possible using the Gamble solution as simulated lung fluid (pH = 4 and at body temperature) so to reproduce the macrophage phagolysosome environment. The investigated mineral fibres display very different dissolution rates. For a 0.25 μm thick fibre, the calculated dissolution time of chrysotile is in the range 94–177 days, very short if compared to that of amphibole fibres (49–245 years), and fibrous erionite (181 years). Diffraction and SEM data on the dissolution products evidence that chrysotile rapidly undergoes amorphization with the formation of a nanophasic silica-rich fibrous metastable pseudomorph as first dissolution step whereas amphibole asbestos and fibrous erionite show minor signs of dissolution even after 9–12 months.

## Introduction

Chrysotile, amphibole asbestos and erionite are the most relevant and widespread mineral fibres included by the International Agency for Research on Cancer (IARC) in Group 1 as Carcinogen (mainly mesothelioma) for humans^1,2^.

The group of amphibole asbestos includes five species: actinolite asbestos Ca_2_(Mg,Fe)_5_Si_8_O_22_(OH)_2_, tremolite asbestos Ca_2_Mg_5_Si_8_O_22_(OH)_2_, anthophyllite asbestos (Mg,Fe^2+^)_7_Si_8_O_22_(OH)_2_, crocidolite (fibrous variety of riebeckite) Na_2_(Fe^2+^,Mg)_3_Fe_2_^3+^Si_8_O_22_(OH)_2_ and amosite (Fe^2+^,Mg)_7_Si_8_O_22_(OH)_2_. Amphiboles are double-chain silicates with a Si(Al):O ratio of 4:11. The oxygen atoms of the chains can coordinate Si(Al) and a variety of other cations; the simplified general formula for amphiboles is^3^: A_0–1_B_2_C_5_T_8_O_22_W_2_. The anions W (OH, F, Cl, O^2−^) occur at the O(3) site, T (Si^4+^, Al^3+^) are the tetrahedrally coordinated sites within the silicate chain, the C cations (Mg^2+^, Fe^2+^, Mn^2+^, Al^3+^, Fe^3+^, Ti^3+^, Ti^4+^, Li^+^, Mn^3+^) occur at the octahedrally coordinated sites *M*(1), *M*(2) and *M*(3) in monoclinic amphiboles, the B cations (Na^+^, Li^+^, Ca^2+^, Mn^2+^, Fe^2+^, Mg^2+^) occur at the 8-fold coordinated *M*(4) site in monoclinic amphiboles, and the A cations (Na^+^, K^+^, Ca^2+^, Li^+^) occur in the A cavity with coordination number from 6 to 12.

Chrysotile is the most common asbestos mineral. It is a trioctahedral hydrous layer silicate based on a 1:1 layer structure with a Si-centred tetrahedral sheet and a Mg-centred octahedral sheet. The ideal chemical formula is Mg_3_Si_2_O_5_(OH)_4_. The lateral dimension of an ideal octahedral Mg-centred sheet (*b* = 9.43 Å) is larger than the lateral dimension of an ideal Si-centred tetrahedral sheet (*b* = 9.1 Å). To a first approximation, this misfit is partly overcome by the curvature of the layer which results in an overall cylindrical lattice^4^.

Erionite is a widespread zeolite whose framework is composed of columns of cancrinite cages^5,6^ connected by a double six-membered ring of tetrahedra, forming hexagonal prisms. Its fibrous form has usually diagenetic origin and occurs in volcanic tuffs.

Although these mineral fibres have all been classified as carcinogens for humans, they are still subject of intensive investigations as the mechanisms by which they induce cyto- and geno-toxic damage are not fully understood to date and comprehensive and shared quantitative figures on their toxicity potential have not been drawn yet. Interpretation of the toxicity and carcinogenicity data is made ambiguous by the very nature of mineral fibres: they are natural materials with great variability in their chemistry, molecular arrangement, size and diameter, surface activity, ability to generate reactive oxygen species and biopersistence^7,8^ so that devising a quantitative model from the data existing in the literature is nearly impossible. Because of this grey area in the scientific knowledge, to date all amphibole asbestos minerals are banned worldwide whereas chrysotile is banned only in the countries where the line of the IARC and World Health Organization (WHO) has been fostered^9^.

In view of drawing a general model of toxicity of mineral fibres and shed light on the global issue of chrysotile, a granted long term Italian Research Project of National Interest (PRIN) is in progress since 2011. Supported by a strong chemical-physical, mineralogical and structural characterization of the fibres, the ultimate goal of this multidisciplinary project is to draw a quantitative model of toxicity of mineral fibres.

One of the key parameters in the model of fibres’ toxicity is biopersistence as it influences the long-term toxicity (the longer a fibre persists in the lower respiratory tract, the greater is the likelihood that it will cause adverse effects) and pathogenicity in the lungs. Biopersistence is defined as the ability of a fibre (or particle in general) to be biodurable (to persist in the human body to physico-chemical processes such as dissolution, leaching, breaking, splitting) and to survive physiological clearance^10–12^. Both *in vitro* techniques to determine fibre biodurability at different pHs and *in vivo* tests to determine the overall biopersistence in the lungs have been developed^10^. *In vitro* tests measure only dissolution rates R of the fibres (biodurability) whereas *in vivo* methods measure the overall retention behaviour of the fibre in the lungs (biopersistence). Comprehensive descriptions of the concepts of biopersistence and biodurability applied to both mineral and synthetic fibres can be found in the specific literature^10–15^. There are two families of *in vitro* tests: (i) cellular *in vitro* investigation includes the treatment of cultured cells with fibres, followed by microscopic examination of the intracellular fibres to determine the change in their diameter and composition. For cellular dissolution tests, alveolar macrophages are commonly used^16,17^. It must be pointed out that the cellular systems have a number of limitations, since the cells are not in their natural environment and the volumes of used media are small compared to *in vivo* systems^18^; (ii) acellular *in vitro* tests, generally conducted by leaching of specific fibre constituents into the dissolution medium (e.g. simulated lung fluids = SLF), are performed both at pH = 4–4.5 and pH = 7.4 simulating the intracellular phagolysosome^19^ and extracellular milieu^20^, respectively. Experiments can be conducted in batch or flow-through reactors. A batch reactor is an inert container where a known mass of fibres is in contact with a fixed volume of fluid. Buffer reagents may be added to the solution to stabilize the pH. In flow-through cells, the dissolution rate R is measured under fixed saturation state conditions by modifying flow rate, initial sample mass and concentration of input solution^12^. Dissolution experiments are performed at 37 °C to simulate human body temperature, using either static or dynamic methods. The degree of dissolution is determined by measuring the change of the sample mass of fibres, and/or the concentration of the ions released into the simulated body fluid^21,22^.

Although *in vitro* experiments cannot substitute *in vivo* experiments due to the complexity of the human body and the multitude of processes than may occur, they provide a benchmark to estimate the biological breakdown of the fibres.

Regarding *in vitro* acellular dissolution studies, earlier works^23,24^ demonstrated that chrysotile dissolves faster than anthophyllite and tremolite asbestos, along the entire pH scale (R = dissolution rate, R_chr_ > R_ant_ >> R_trm_).

Regarding dissolution in mimicked lung fluids, the literature reports only a few case studies. Hume and Rimstidt^25^ (1992) demonstrated that the dissolution of chrysotile is controlled by the dissolution rate of the silica layer. Oze and Solt^22^ measured the dissolution rates of chrysotile and tremolite in batch reactors using simulated lung (pH 7.4) and gastric (pH 1.2) fluids. Rozalen *et al*.^23^ measured the dissolution of tremolite at 37 °C using simulated macrophages (pH 4) and interstitial fluids (pH 7.4).

Although it is hard to compare the results of *in vitro* biodurability studies to the ones obtained by *in vivo* biopersistence studies^10^, the general trend in the literature is that the rate of dissolution both *in vitro* and *in vivo* of chrysotile is higher than that of amphibole asbestos. Studies on rats^26^ showed that the rate of clearance of chrysotile was three times higher than that of amphiboles. A higher rate of clearance of chrysotile with respect to amphibole asbestos was confirmed^27^. In contrast, Middleton *et al*.^28^ found no difference in the asbestos clearance rate related to the fibre type, even if the observed retention of chrysotile was lower compared to that of amosite and crocidolite. The clearance studies on UICC (Union for International Cancer Control) amosite by Bolton *et al*.^29^ confirmed previous findings^28^. Later studies on asbestos exposure of populations and workers evidenced a preferential retention in the lung of amphiboles compared to chrysotile^30,31^. Jaurand *et al*.^16,17^ observed a progressive leaching of Mg from the chrysotile fibres, leading to a decrease of the Mg/Si ratio over time, as a consequence of the biodegradation of the fibres in biological medium.

Another comparative *in vitro* study on the biodurability of naturally occurring silicate fibres^32^ in simulated extra cellular fluid under flow conditions showed that erionite is much more biopersistent than both crocidolite and chrysotile.

Earlier experiments of intratracheal injection of asbestos in rats^33^ measured a clearance half time longer than 300 days (d) for crocidolite fibres. The persistence of amosite fibres increased with increasing fibre length, with no evidence of clearance for the length class containing fibres longer than 20 µm^14,34^.

Bernstein *et al*.^35^ published inhalation biopersistence studies on different chrysotile asbestos, using a standard biopersistence protocol. In all these studies, chrysotile was found to be less persistent than amphibole asbestos.

The survey of the literature presents a myriad of studies on biodurability of selected mineral fibres using different, sometimes poorly characterized samples and different experimental conditions that prevent a direct comparison. Despite the large amount of literature data, a systematic study of the dissolution of mineral fibres is still missing to date. The present work fulfils this gap and reports a systematic comparative *in vitro* acellular study of the biodurability of the most relevant mineral fibres (chrysotile and amphibole asbestos as well as erionite) aimed at assessing the role of this parameter on their potential toxicity. Gamble solution as simulated lung fluid (SLF) buffered at pH = 4 was used to reproduce the macrophage intracellular phagolysosome chemical environment mimicking the phagocytosis process inside the alveolar space.

## Results

**Figure 1:**
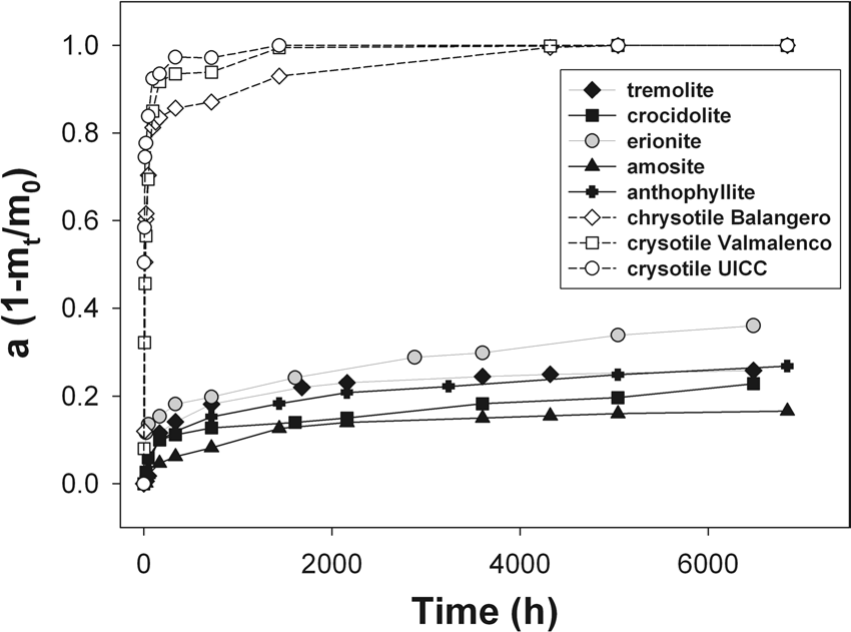
The raw dissolution curves of all the investigated fibres (m_0_ and m_t_ are the fibre mass at time 0 and t, respectively).

The raw dissolution curves of all the investigated fibres are plotted in Fig. 1. The difference in the dissolution time of chrysotile asbestos with respect to amphibole asbestos and fibrous erionite is striking. After about 4000 h, the chrysotile samples are completely dissolved. On the other hand, after the same time span, the amount of amphiboles species and erionite that is dissolved is lower than 30%. Among the chrysotile species, the dissolution of the UICC sample is faster than the others with the Balangero chrysotile exhibiting the slowest dissolution rate. Among the other mineral fibres, the dissolution of erionite is faster than the amphibole species. Tremolite and anthophyllite asbestos dissolve faster than the iron rich species crocidolite and amosite with amosite showing the slowest dissolution rate. Distinct dissolution curves for chrysotile, fibrous erionite and fibrous amphibole are deposited as Supplementary Material.

Table 1 reports the BET specific surface area (SSA_BET_) and the kinetic parameters calculated from the fit of the data following the procedure described in the experimental part together with the regression coefficients. In detail, the table contains the apparent rate constant k, the apparent dissolution rate R, logR, and the estimated lifetime t of a fibre (in days and years).

**Table 1:** Kinetic parameters calculated for the dissolution of mineral fibres.

**Sample**	**SSA**_**BET**_ **(m** ^**2**^ **g** ^**−1**^ **)**	**k (s** ^**−1**^ **)**	**R** ^**2**^	**R (mol·m** ^**−2**^ **s** ^**−1**^ **)**	**logR**	**t** ^**ℵ**^ **(days)**	**t** ^**ℵ**^ **(years)**
UICC amosite	9.5(3)	6.1(6) × 10^−14^	0.999	2.7(3) × 10^−13^	−12.57	27010(2647)	74(7)
UICC anthophyllite asbestos	4.4(2)	1.2(3) × 10^−13^	0.996	1.0(3) × 10^−13^	−13.00	83950(20990)	245(64)
UICC crocidolite	16.1(6)	1.3(3) × 10^−13^	0.990	3.2(7) × 10^−13^	−12.49	24090(5840)	66(16)
*Val d’Ala* tremolite asbestos	9.2(3)	5.4(9) × 10^−14^	0.933	4.5(7) × 10^−13^	−12.35	17885(2981)	49(8)
fibrous erionite	372(11)* 39(2)** 12.7(5)***	2.4(9) × 10^−14^	0.975	2.4(9) × 10^−14^	−13.62	62415(24774)	181(68)
Balangero chrysotile	42(1)	1.8(6) × 10^−10^	0.838	1.7(6) × 10^−10^	−9.76	124(41)	0.3(1)
UICC chrysotile	43(2)	2.5(7) × 10^−10^	0.842	2.3(6) × 10^−10^	−9.64	94(26)	0.3(1)
Valmalenco chrysotile	68(9)	2.1(6) × 10^−10^	0.914	1.2(3) × 10^−10^	−9.91	177(51)	0.5(1)

^*^SSA_BET_ of the sample dehydrated at 400 °C for 1 night; **t-Plot external surface area of the sample dehydrated at 400 °C for 1 night;

***SSA_BET_ of the natural sample; ^ℵ^calculated for a 0.25 μm thick fibre.

## Discussion

**Figure 2:**
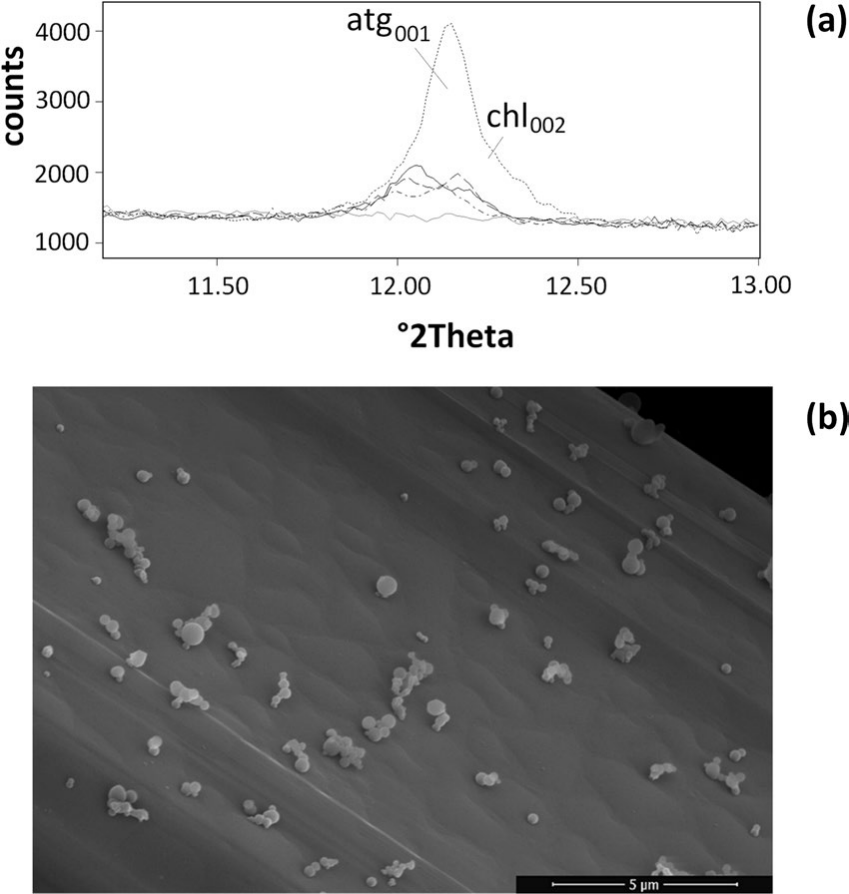
(**a**) X-ray powder diffraction (CuKα radiation) data of asbestos tremolite in the region including the major peaks of the impurities antigorite (atg_001_) and chlorite (chl_002_), witnessing an early dissolution of these phases during the first 2 months of the experiment. Legend: short dashed line = 24 h; black solid line = 48 h; long dashed line = 336 h; dashed and dots = 720 h; gray solid line = 1440 h; (**b**) Ca-rich precipitate (likely portlandite) at the surface of tremolite fibres formed during the dissolution experiment.

For each fibre sample, our procedure of analysis permitted to accurately determine not only the weight of the solid product of dissolution with time (gravimetry), but also the weight fraction of each crystalline and amorphous phase in the solid residue using Full quantitative phase analysis (FQPA). With this approach, bias due to the differential dissolution of impurities eventually present in the samples has been taken into account. In the tremolite asbestos sample for example, early dissolution of the Si- and Mg-rich impurities antigorite and clinochlore has been observed (Fig. 2a). By monitoring the release of Si and Mg from tremolite, the dissolution rate would have been biased by the early dissolution of these impurities and a faster dissolution rate would have been found. Correctly, the determination of the weight fraction of each phase by the FQPA in the reacting system allowed to estimate the actual residual weight of tremolite asbestos and the impurities which dissolves with time and to calculate an unbiased dissolution rate. If precipitation occurs during dissolution, the rate based only on elemental dissolution or weight is again biased. With the FQPA, the presence of crystalline or amorphous precipitates can be detected and quantified to correctly estimate the actual weight of the fibre species in the reacting system. Tremolite asbestos is used again as example: high resolution SEM imaging revealed the precipitation of a Ca-rich phase (possibly portlandite Ca(OH)_2_) at the surface of the fibres (Fig. 2b). If this phase is not taken into account, the estimate of the dissolution rate using both the overall weight of the solid residue or monitoring of Ca release in solution is biased.

As described in the experimental part, the apparent dissolution rate (R) was normalized with respect to the specific surface area (SSA) for the slow-dissolving fibre minerals, which is a fair approximation of the reactive surface area considering the short time-span of the experiment with respect to the lifetime t of the fibres (Table 1). This is not the case for chrysotile that reaches complete dissolution. Considering that the area of the reaction front controls the dissolution kinetics, the experimental dissolution data must be related to the change in reactive surface area which in turn corresponds to the advancement of the Mg leaching front (see later discussions) in the case of chrysotile. Because of the difficulties in directly measuring this property, geometrical models are generally applied^36^. The model used in this work allowed us to calculate the corrosion distance (*x*_*t*_) and consequently the remaining surface fraction with time, assuming a fibre population composed of equally sized fibres and using SSA_BET_ or external surface area and density (ρ) as input parameters. A similar model was used by others^36^, although the length-weighted distribution of the fibre diameter was used as input parameter instead of SSA. It should be noted that Guldberg *et al*.^36^ mainly investigated man-made vitreous fibres (MMVF) with very broad size distribution which motivated this choice. An approximation of our model is that we did not consider the case of fibres having a log normal distribution.

**Figure 3:**
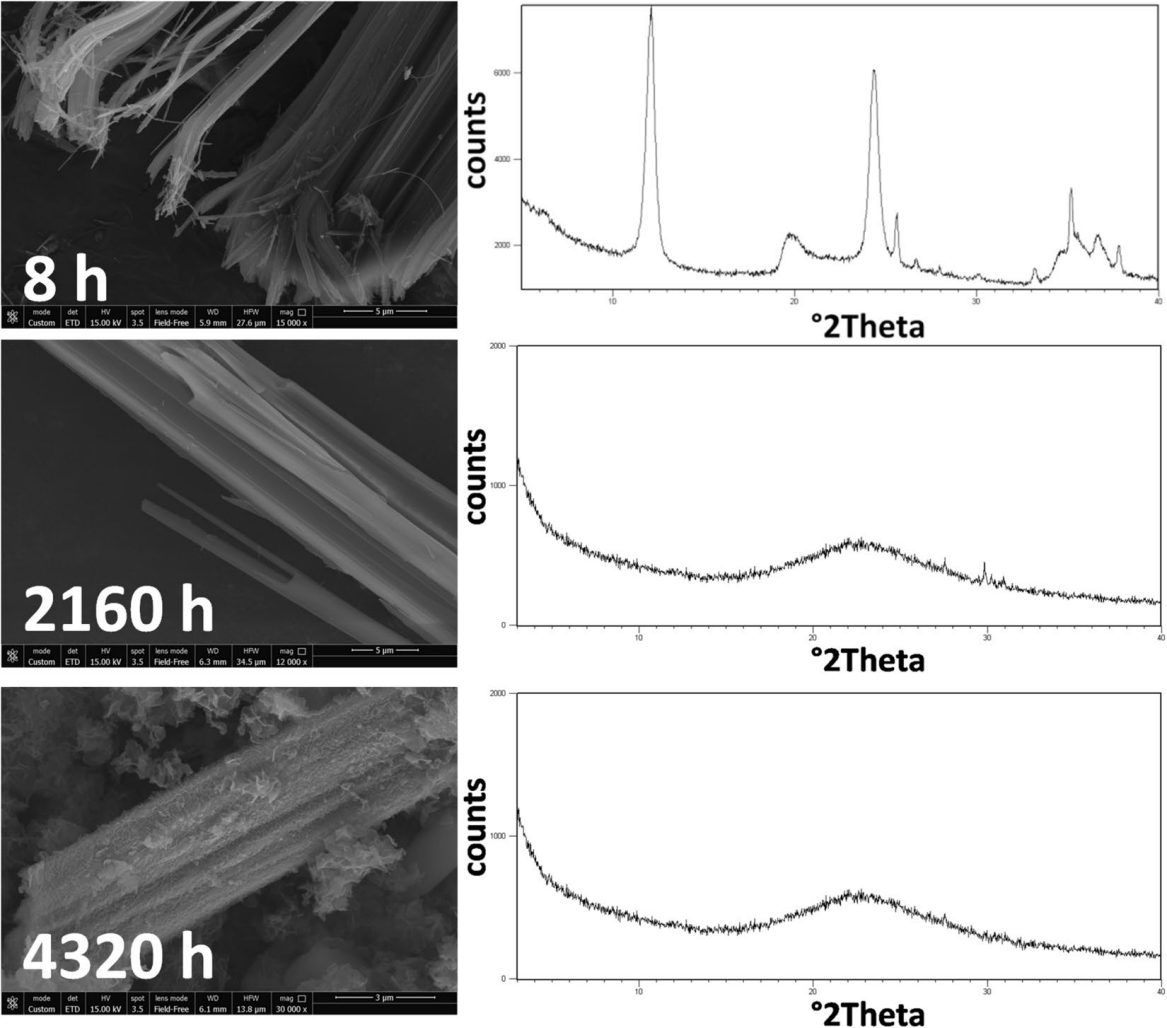
FEG-SEM images and X-ray diffraction traces of the UICC chrysotile fibres during the dissolution experiment: top = after 8 h; middle = after 3 months; bottom = after 6 months.

**Figure 4:**
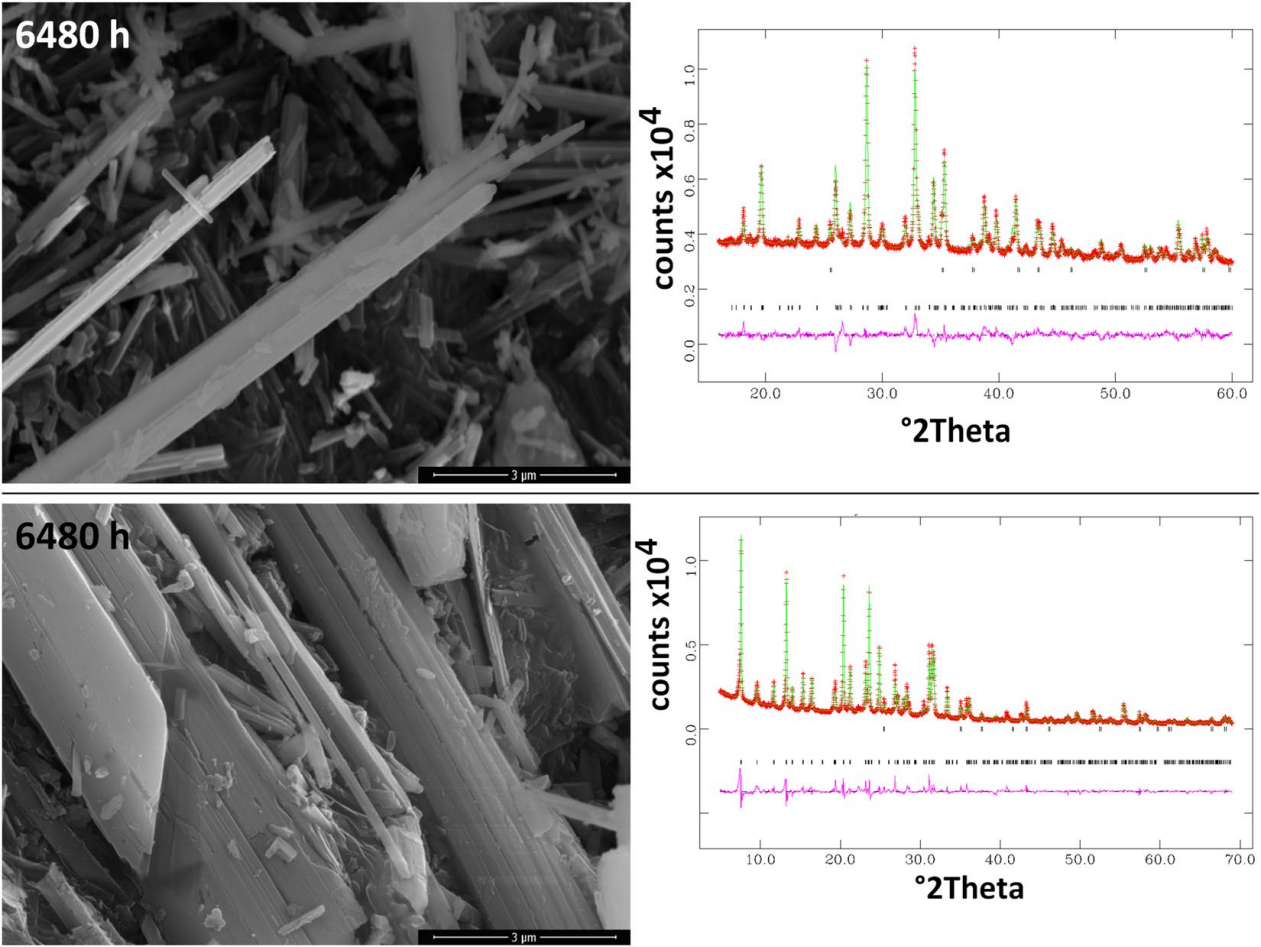
FEG-SEM images and the graphical outputs of the X-ray diffraction Rietveld refinements of crocidolite and fibrous erionite solid residue of the dissolution experiment collected after 9 months. Each graphical output reports the points of observed pattern (red crosses), the calculated pattern (green line), and the difference curve (observed pattern - calculated pattern) below. Markers with the positions of the peaks of each phase in the system are also reported.

In concert with the findings of this work, recent results^37–39^ have demonstrated that chrysotile reacts much faster than crocidolite and fibrous erionite-Na both *in vitro* (simulated lung fluids SLF and inorganic media) and *in vivo*. Chrysotile fibres undergo Mg leaching (as demonstrated by the chemical analysis on the solid reaction products that no longer contain Mg) and form fibrous amorphous silica relicts. On the other hand, crocidolite and erionite-Na show minor signs of amorphization^38,39^ with the formation of a nanometer thick silica-rich surface amorphous layer. The X-ray diffraction (CuKα radiation) and electron microscopy data on the dissolution products witness that chrysotile readily undergoes amorphization as a first dissolution step whereas amphibole asbestos fibres and fibrous erionite show only minor loss of crystallinity even after 9–12 months. Figure 3 shows UICC chrysotile fibres during the dissolution experiment (after 8 h, 3 months and 6 months). The X-ray (CuKα radiation) patterns of the solid residue collected after 3 and 6 months evidence the amorphous nature of the metastable product whereas FEG-SEM images show that the overall crystal habit is preserved (‘pseudomorphosis’ phenomenon). Comparable results are found for Balangero and Valmalenco chrysotiles. Both amphibole asbestos fibres and fibrous erionite are mostly crystalline even following longer dissolution times, showing a well preserved crystal habit and sharp X-ray diffraction peaks (see for example, crocidolite and erionite after 9 months in Fig. 4).

Although, only a few studies of the dissolution kinetics of mineral fibres are available in the literature, they are in agreement with the dissolution rates calculated from our work (Table 1). As far as anthophyllite is concerned, the dissolution rate R^40^ based on Mg dissolution at 25 °C and pH = 3.56 of 1.6 × 10^−13^ mol·m^−2^s^−1^ is in agreement with our figure (1.0(3) × 10^−13^ mol·m^−2^s^−1^ in Table 1). There is agreement also with Rozalen *et al*.^24^ (logR = −11 at pH = 4). The same applies to tremolite^24^: logR = −13 at pH = 4. Concerning chrysotile, dissolution rates (Table 1) are in the range 1.7–2.3 × 10^−10^ mol·m^−2^s^−1^, in agreement with Hume and Rimstidt^25^ (4.38–6.27 × 10^−10^ mol·m^−2^s^−1^ for chrysotile dissolution rates at pH = 4). Accordingly, the total estimated dissolution times are comparable: 0.75 years – no error reported - for a 1 μm thick fibre^25^ and from 0.3(1) to 0.5(1) years for a 0.25 μm thick fibre (our work). Our data are in line with the findings of Parry^41^ but both literature data and our results are in disagreement with Oze and Solt^22^ who postulated a surprisingly long lifetime of chrysotile (157 years) and short for tremolite (12 years) – no error reported - assuming 1 μm thick particles dissolving in SLF. A possible source of bias in the calculated lifetimes reported by Oze and Solt^22^ is that the rate constants were not normalized with respect to the surface areas of the fibres.

Chrysotile asbestos, amphibole asbestos and fibrous erionite display different chemistry and structural arrangements reflecting in different dissolution rates. Concerning the dissolution mechanism, both literature data and the results of our experiments point to similar overall processes. Dissolution of chrysotile fibres is known to proceed by two steps: decomposition of the octahedral layer with Mg leaching (substitution of Mg^2+^ for H^+^ or H_3_O^+^), and silica layer dissolution at a slower rate. Hence the lifetime of the chrysotile fibre is controlled by the dissolution rate of the silica layer^25^.

For the amphibole species like tremolite, the postulated dissolution model also predicts replacement of Mg^2+^ for H^+^ in the mineral structure during hydrolysis, without a major change in the silica network. In fact, cation depletion on the surface of tremolite results from the substitution of Ca^2+^ and/or Mg^2+^ for H^+^ (or H_3_O^+^) in a modified amorphous structure^42^. Silica dissolution is also the “rate limiting step” with the formation of an amorphous layer eat the surface of tremolite (3 nm thick after 24 days at pH = 1 and 20 °C)^42^.

In line with previous results^37,38^, this study confirms that the dissolution of fibrous erionite also involves the formation of an early surface reaction layer consisting of amorphous silica. This layer is depleted in both Al and extraframework cations although the overall structure is preserved^43^. The structure of HCl-treated erionite does not collapse but a porous amorphous surface layer is formed after decationization and dealumination^43^. Dissolution via sequential removal of surface layers has also been observed in the past for other zeolite species like heulandite^44^. It has been observed that zeolite dissolution rate via dealumination can result in stoichiometric framework degradation, silicate precipitation, partially dissolved silicate framework, or intact silicate framework dependent upon the initial Si/Al ratio^45^. Acid zeolites like erionite with Si/Al ratio >2.5 show little to null dissolution in acidic environment because dealumination does not eventually cause the collapse of the framework.

**Figure 5:**
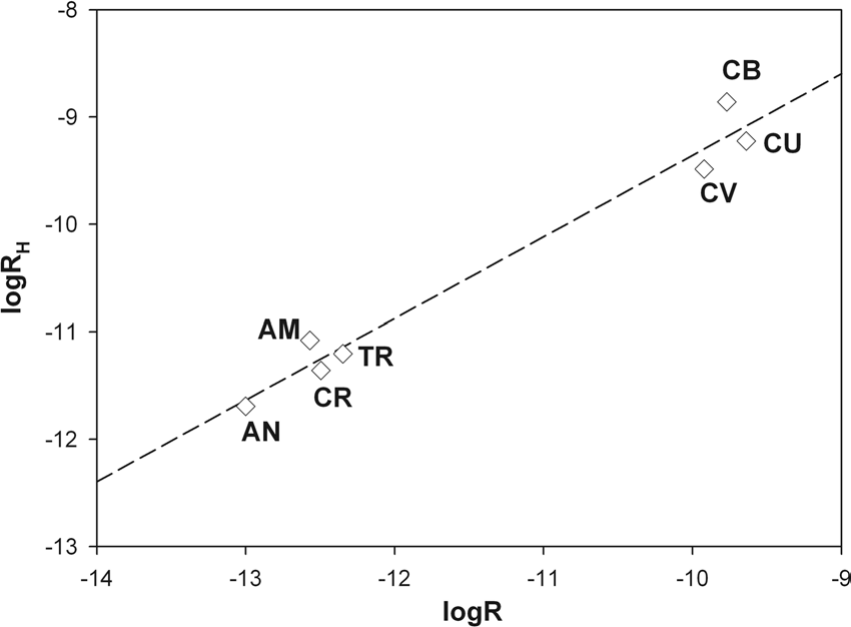
Plot of the ideal logR_H_ values calculated using the k_H_ predicted for chrysotile and amphiboles using the eq. reported in Brantley^46^ vs. the logR values calculated from our dissolution experiments (see the text for details). Legend: AM = UICC amosite; AN = UICC anthophyllite asbestos; CR = UICC crocidolite; TR = *Val d’Ala* tremolite asbestos; CB = Balangero chrysotile; CU = UICC chrysotile; CV = Valmalenco chrysotile.

For the amphiboles (inosilicates) and the chrysotile (phyllosilicate) families, prediction of the rate constant k_H_ (at 25 °C) has been accomplished using the equation (1)^46^:

1 $${{\rm{k}}}_{{\rm{H}}}=\frac{{10}^{13.51\mathrm{log}(0.5\frac{{\rm{X}}}{{\rm{Si}}})+1.22{{\rm{logk}}}_{{\rm{solv}}}-13.6}}{{\upsilon }_{{\rm{Si}}}}$$

where $\frac{{\rm{X}}}{{\rm{Si}}}$ = ratio between non-tetrahedral cations versus tetrahedrally coordinated cations (Si,Al); k_solv_(s^−1^) = chemical species dependent rate constant of solvent exchange; ${\upsilon }_{{\rm{Si}}}$ = number of Si in the mineral unit formula (ex. 4 for ideal chrysotile and 8 for ideal amphiboles). With the chemical/structural parameters from the formula of the investigated mineral fibres, k_H_ values can be calculated and converted into R_H_ values using eq. (1) where SSA (m^2^·g^−1^) is the measured specific surface (Table 1) and the mass is 0.025 g. The predicted logR_H_ values are plotted vs. the dissolution rates logR calculated from the kinetic experiments (Fig. 5). The agreement between the predicted and experimental rates is indicative of the accuracy of our kinetic model. Erionite is a framework silicate and cannot be included in the prediction model. As far as the dissolution rate is concerned, Fig. 5 clearly shows two distinct families, one with a fast dissolution rate (chrysotile asbestos) and the other with a slow dissolution rate (amphibole asbestos). The discriminating chemical parameters is $\frac{{\rm{X}}}{{\rm{Si}}}$, the ratio between non tetrahedral cations (mostly Mg) versus tetrahedrally coordinated cations (mostly Si), which is ideally 0.875 for amphiboles and 1.5 for chrysotile. The differences among the samples of the chrysotile asbestos family are negligible whereas significant differences are observed for the amphibole asbestos family. These differences are obviously due to the nature of the non-tetrahedral cations (Mg, Fe, Ca, Na) and their solvent (H_2_O) exchange affinity measured in terms of k_solv_^47^.

**Figure 6:**
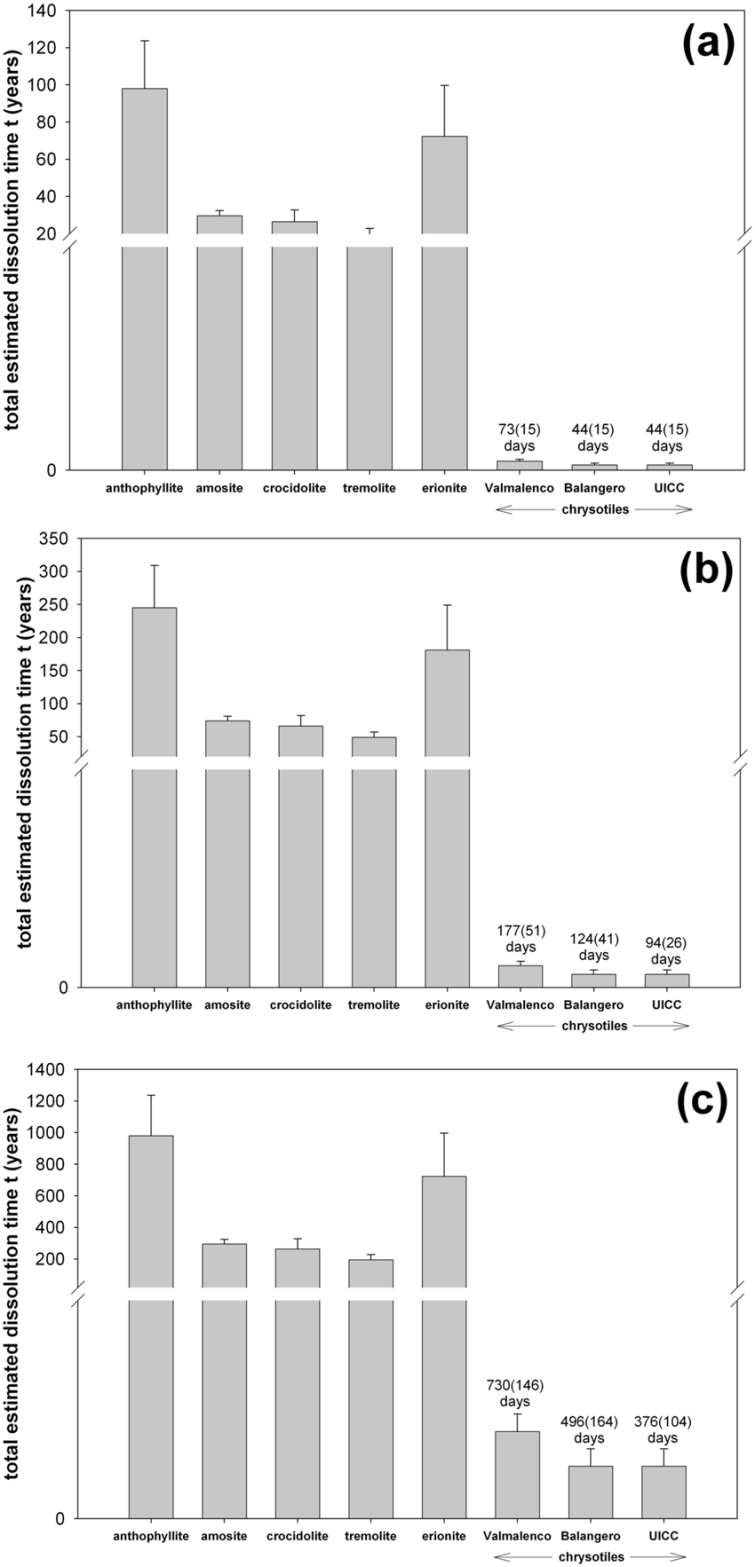
Prediction of the total dissolution time of each investigated mineral assuming a fibre diameter of 0.1 μm (**a**), 0.25 μm, and 1 μm.

Although there have been many studies of *in vitro* durability on selected mineral fibres, this is the first time a systematic comparative study of the *in vitro* biodurability of chrysotile, amphibole asbestos and fibrous erionite is performed. Figure 6 and Table 1 summarize the results of our study. For a fibre diameter of 0.25 μm, the calculated dissolution time of chrysotile spans from 94(31) to 177(35) days. This time is very short if compared to that of the amphibole mineral fibres that spans from 49(8) to 245(64) years. Erionite with a fibre diameter of 0.25 μm has a total dissolution time of 181(68) years (Table 1). Figure 6 reports the total dissolution times of fibres having the diameters 0.1 μm (a), 0.25 μm (b), and 1 μm (c). These results unequivocally confirm previous findings postulating a remarkable difference in both biodurability *in vitro* and clearance *in vivo* of chrysotile with respect to amphibole asbestos. Chrysotile lies towards the soluble end of the scale with a fast clearance from the lungs (clearance half time for 5–20 μm long fibres is the range 2.4–29.7 – no error reported - days)^11,48,49^. On the other hand, amosite, crocidolite, and tremolite asbestos have very slow clearance times from the lungs (clearance half time for 5–20 μm long fibres is 900 days for amosite, 262 days for crocidolite – no error reported - and infinite (?) for tremolite)^11,14^. An early acid dissolution study^50^ also showed that chrysotile is not biodurable but surprisingly it was reported that tremolite is apparently much more biodurable than amosite. Figure 5 in Bernstein *et al*.^49^ shows the clearance time of chrysotile, amosite and tremolite from the rat lung, evidencing lack of biopersistence for chrysotile and comparable biopersistence for amosite and tremolite, in agreement with our study.

Although biodurability should not be considered synonymous of biopersistence and the fact that there are still open questions about the correlation between the results *in vitro* and *in vivo*^36^, this parameter is taken into account to assess the toxicity potential of asbestos and ‘man-made’ mineral fibres. Made this caveat, our results would support a model of lower toxic potential for chrysotile with respect to the amphibole asbestos and fibrous erionite.

Among biodurable amphibole asbestos fibres, anthophyllite is by far the most biodurable species. This information can be useful to assess the actual toxicity of this fibre species as its toxicity has not been well studied compared to other asbestos types^51^. Some authors claimed that when it comes to inducing mesothelioma, anthophyllite is less potent than other amphibole asbestos and possibly even chrysotile^52^. On the contrary, anthophyllite is found to be more effective than chrysotile in inducing mesothelioma^53^. If biodurability is assumed to be a relevant toxicity parameter (see above), the results of our study certainly support the view that the potential toxicity of anthophyllite is much higher than that of chrysotile.

Although the results of our work quantitatively demonstrate that chrysotile asbestos is much less biodurable than amphibole asbestos and fibrous erionite, during the dissolution process, chrysotile produces a metastable fibrous amorphous silica^54^ in a process classified as ‘pseudo-morphosis’^38^. The process later leads to the physical destruction of the fibres^35,49^. The activity of this fibrous silica residue *in vitro* and *in vivo* is unknown to date and work is in progress to study its structural-chemical characters and potential toxicity/pathogenicity.

## Methods

### Materials

The following mineral fibres have been selected for the study:

1. UICC standard amosite (South African, NB #4173–111–4) [(Ca_0.02_Na_0.01_)(Fe^2+^_5.36_Mg_1.48_Fe^3+^_0.11_Mn_0.06_)_7.01_(Si_7.93_Al_0.01_)_7.94_O_21.94_(OH)_2.06_] with minor impurities (<1 wt%) of calcite, hematite and quartz. Mean fibre length of 7(2) μm. Molar weight M of amosite is 1001.61 g·mol^−1^, molar volume V is 2.98 × 10^−4^ m^3^·mol^−1^.
2. UICC standard anthophyllite asbestos (Finnish NB #4173-111-5) [Ca_0.04_(Mg_5.81_Fe^2+^_0.92_Fe^3+^_0.21_Mn_0.04_)_6.98_(Si_7.83_Al_0.02_)_7.85_O_21.63_(OH)_2.37_]. The sample is contaminated with biotite (1.4(2) wt%), clinochlore/vermiculite (1.7(3) wt%) and talc (7.7(4) wt%). Mean fibre length of 4.4(2) μm. Molar weight M of anthophyllite is 780.82 g·mol^−1^, molar volume V is 2.43 × 10^−4^ m^3^·mol^−1^.
3. UICC standard crocidolite (South African, NB #4173-111-3) [(Na_1.96_Ca_0.03_K_0.01_)_2_(Fe^2+^_2.34_Fe^3+^_2.05_Mg_0.52_)_4.91_(Si_7.84_Al_0.02_)_7.86_O_21.36_(OH)_2.64_] with minor (<1 wt%) impurities of hematite, magnetite, and quartz. Mean fibre length of 6(1) μm. Molar weight M of crocidolite is 935.9 g·mol^−1^, molar volume V is 2.81 × 10^−4^ m^3^·mol^−1^.
4. Tremolite asbestos from Val d’Ala, Turin (Italy) [(Ca_1.91_Na_0.06_K_0.01_)_1.98_(Mg_4.71_Fe^2+^_0.22_Fe^3+^_0.08_Mn_0.02_)_5.03_(Si_8.01_Al_0.02_)_8.03_O_22.14_(OH)_1.86_] with impurities of antigorite (5.1(5) wt%) and clinochlore (2.4(4) wt%). Mean fibre length of 11(1) μm. Molar weight M of tremolite is 812.37 g·mol^−1^, molar volume V is 2.73 × 10^−4^ m^3^·mol^−1^.
5. Balangero (Torino, Italy) chrysotile [(Mg_5.81_Fe^2+^_0.15_Al_0.27_Fe^3+^_0.09_Cr_0.01_)_6.33_Si_3.97_O_10_(OH)_7.11_] with impurities of antigorite, balangeroite, calcite, clinochlore, diopside, dolomite, magnetite, microcline, plagioclase, talc. Mean fibre length of 6(1) μm. Molar weight M is 277.11 g·mol^−1^, molar volume V is 1 × 10^−4^ m^3^·mol^−1^.
6. UICC standard chrysotile “B” asbestos [(Mg_5.93_Fe^2+^_0.04_Al_0.02_Fe^3+^_0.08_)_6.07_Si_4.03_O_10_(OH)_7.66_] with impurities of brucite, calcite, clinochlore, dolomite, magnetite, microcline, pyroaurite and talc. Mean fibre length of 5(2) μm. Molar weight M is 277.11 g·mol^−1^, molar volume V is 1 × 10^−4^ m^3^·mol^−1^.
7. Valmalenco (Sondrio, Italy) chrysotile (Mg_5.85_Fe^2+^_0.06_Al_0.02_Fe^3+^_0.05_Ni_0.01_)_5.99_Si_4.01_O_10_(OH)_7.86_] with impurities of calcite, forsterite, magnetite, quartz, lizardite/antigorite, clinochlore. Mean fibre length of 10(5) μm. Molar weight M is 277.11 g·mol^−1^, molar volume V is 1 × 10^−4^ m^3^·mol^−1^.
8. Erionite-Na from Jersey, Nevada (USA) [(Na_5.35_K_2.19_Ca_0.15_Mg_0.11_Ti_0.05_)_7.85_(Si_28.01_Al_7.90_)_35.91_O_72_∙28.13H_2_O] with traces of clinoptilolite. Mean fibre length of 12.5(5) μm. Molar weight M of erionite is 2809.97 g·mol^−1^, molar volume V is 1.36 × 10^−3^ m^3^·mol^−1^.

It should be remarked that the UICC samples were ground in large industrial milling machines and do not necessarily represent the fibres as used commercially.

The measured specific surface areas (SSA) of the samples are reported in Table 1. A detailed characterization of the mineral fibres described in this work can be found in Pollastri *et al*.^8,55^.

### Dissolution experiments and characterization

To mimic the phagocytosis process inside the intracellular alveolar space, dissolution experiments of the mineral fibres were conducted using the SLF Gamble solution^56^ buffered at pH = 4. Its composition (values expressed in mg/l) is: 106.8 MgCl_2_·6H_2_O, 3208 NaCl, 72 Na_2_HPO_4_, 40 Na_2_SO_4_, 128 CaCl_2_·2H_2_O, 77.2 C_6_H_5_O_7_·2H_2_O·3Na, 6000 NaOH, 20800 C_6_H_8_O_7_, 59.2 NH_2_CH_2_COOH, 90 C_4_H_8_Na_2_O_8_, 141.6 NaC_3_H_5_O_3_ (60% w/w), and 86 C_3_H_3_NaO_3_. We are aware of the fact that fibres may be located in the extracellular space (pH = 7.4) but their dissolution rate in that environment is too slow, even for chrysotile, to allow an accurate kinetic study. Hence, experiments were not replicated at neutral pH. 5 ml of formaldehyde were added to the SLF to prevent formation of algae/mould. All the dissolution experiments were conducted under the very same conditions, using 250 ml polyethylene bottles (batch reactors) kept in continuous agitation (90 rpm) inside an oscillating bearing incubator kept at 37 °C. For each fibre species, 20 bottles were filled with the buffered SLF and 0.02500 g of sample (previously conditioned at 105 °C for 24 h). A static setup with concentrations of mineral fibres in suspension of 1 × 10^−4^ g/ml was used to employ experimental conditions comparable to those reported in pertinent literature data^22^. It may be argued that at these concentration, the constituent metals of the fibres are above the solubility limits but the opposite is true if one considers that a ratio of 6 × 10^−3^ g/ml is found inside a macrophage when a fibre 0.25 μm thick and 3 μm long is internalized inside a 3 μm thick phagolysosome.

The pH of the solution was monitored during the experiments. At specific times (e.g., 4 and 8 h, 1, 2, 7, and 14 d, 1, 2, 3, 6, 9, and 12 months, and random replicates), the advancement of the dissolution reaction was determined.

In previous studies, the reaction advancement was followed indirectly by determining the concentration of ions in solution (silica or e.g. Mg, Ca, Na, Al) released from the fibres^56^. This experimental strategy works well for pure synthetic samples (such as MMMF) but may lead to artifacts in the event that: (i) the fibres are contaminated with impurities as in the case of natural samples; (ii) a re-precipitation of the silica phase of other elements in the form of gel-like compounds occurs; (ii) silica is not the major component of the system. In this study, we have deliberately chosen to follow the dissolution kinetics by measuring the weight loss of the samples and accurately determine the weight fraction of the mineral fibre in the solid residue. In this way, artifacts due to the presence of pristine impurities and metastable or secondary amorphous/crystalline precipitation products are avoided. We have tested the consistency of the data collected with our method with those collected with the classical method to measure ions released in solution for one of the samples, the Balangero chrysotile and obtained a good match (see the results reported in the Supplementary Material).

The following protocol was applied to each sample: at predetermined sampling times (see above), the content of the bottle was vacuum filtered using conditioned and weighed 0.22 µm ϕ cellulose Merck Millipore filters (ashless grade). The solid residue was flushed with ultrapure distilled water to remove salts from the Gamble solution. The weight of the solid residue was accurately determined by weight difference, following ashing of the filter. Full quantitative phase analyses (FQPA) of the solid residues were performed using X-ray powder diffraction (XRPD) and the Rietveld method^57^. The weight estimates from each investigated sequence are deposited as Supplementary Material. Hence, the actual weight fraction of the pristine fibre minerals was obtained, thus avoiding bias due to impurities and secondary precipitates (as discussed above). XRPD data were collected using a PANalytical Bragg–Brentano θ-θ diffractometer with CuKα radiation, 40 kV and 40 mA, a real time multiple strip (RTMS) detector, soller slit (0.02 rad), ½° divergence and ½° anti-scattering slits mounted in the incident beam pathway, soller slit (0.02 rad), Ni filter and antiscatter blade (5 mm) along the pathway of the diffracted beam. The virtual step size of the measurement was 0.0167° 2θ. FQPA were performed using the Rietveld method^57^ with the General Structure Analysis System (GSAS) package^58^ and its graphical interface EXPGUI^59^. The determination of the actual weight loss of each mineral fibre through the calculation of both crystalline and amorphous content was performed using the combined Rietveld–RIR method^60^.

The solid residues were also observed by scanning electron microscopy (SEM) using a FEI Quanta Nova NanoSEM 450 and a FEI QuantaFEG 450 SEM.

The specific surface area (SSA) of the fibres was determined by the BET-method using a Gemini-V instrument with N_2_ as probe gas. The samples were conditioned at 105 °C prior to the measurement. For the microporous zeolite sample (fibrous erionite), the sample was also conditioned at 400 °C for 1 night prior to the measurement. The total surface area was determined with the BET model whereas the external surface area was determined with the t-plot method.

### Determination of the kinetic parameters

This paragraph reports the basic steps of the procedure used for the analysis of the dissolution data. The detailed description of the kinetic analysis is reported as Supplementary Material.

**Figure 7:**
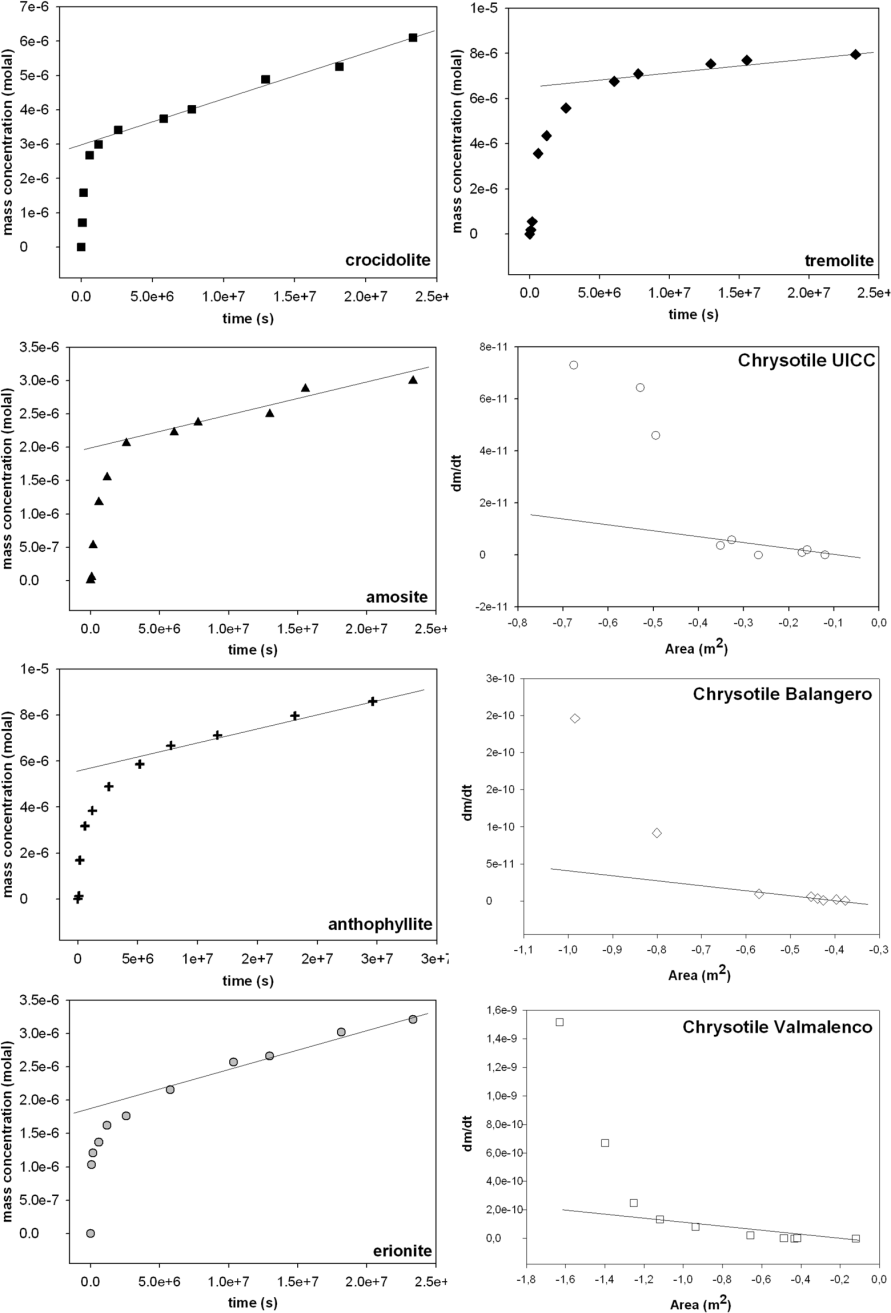
Plots of the molal concentration vs. time for amphibole asbestos fibres and erionite. The fit of the final part of the curve yielded the apparent rate constant k (s^−1^). The second column includes the fit of the dm/dt vs. −A (m^2^) plots for the chrysotile asbestos samples used to determine k from the equation^36^ $\frac{{\rm{d}}{\rm{m}}}{{\rm{d}}{\rm{t}}}{\rm{=}}{\rm{-}}\,{\rm{k}}{\rm{\cdot }}{\rm{A}}({\rm{t}})$. Again, the fit of the final part of the curve yielded the apparent rate constant k (s^−1^) (slope) (see the text for details).

The actual mass of the investigated mineral fibres dissolved with time, accurately determined from the FQPA, was converted into moles (fibre mass/fibre molar weight M) to obtain a plot of moles vs. time (s) like the molal concentration vs. time plots shown in Hume and Rimstidt^25^ (Fig. 7). The fit of the final part of the curve was accomplished with a first order equation that yielded the apparent rate constant k (s^−1^). The calculated value of k was used to determine the apparent dissolution rate R (mol·m^−2^s^−1^) from the equation (2) valid for batch reactors^61^:

2 $${\rm{R}}=\frac{{\rm{k}}}{{\rm{SSA}}\cdot {\rm{m}}}$$

with SSA = measured initial SSA (m^2^/g) (Table 1) and m = initial mass of the sample (g).

In the case of amphibole fibres and erionite, the dissolution rates are very slow and initial SSA is expected to be virtually constant during the experiments. This approximation is acceptable if we consider that the fibre dissolution within the time span of the experiments is limited. For these fibres, the initial measured SSA_BET_ is used as input value in the equation for amphibole asbestos species whereas external surface area is used in the erionite case. This assumption does not apply to chrysotile asbestos fibres completely dissolving during the time span of the experiments. For chrysotile, a geometric model was especially developed (see the Supplementary Material). The model permits to calculate the variation of A (m^2^) with time, used to determine the apparent rate constant k from the equation (3)^36^:

3 $$\frac{{\rm{dm}}}{{\rm{dt}}}=-\,{\rm{k}}\cdot {\rm{A}}({\rm{t}})$$

In this way, the rate constant also includes the contribution of the variation of surface area with time. k is then used to determine the apparent dissolution rate R from the equation above^61^. The fit for the three chrysotile sample is shown in Fig. 7.

For all the fibres, the estimated lifetime t of a fibre was determined using the equation (4)^23,25^:

4 $${\rm{t}}=\frac{3{\rm{d}}}{4{\rm{VR}}}$$

where d = fibre diameter (m): a diameter of 0.25 µm (0.25 × 10^−6^ m) was assumed for the calculation; V = molar volume (m^3^·mol^−1^), R = apparent dissolution rate normalized to the surface area (see above).

## Electronic supplementary material

Supplementary Material

## References

[1] IARC (International Agency for Research on Cancer) (2012). Asbestos (chrysotile, amosite, crocidolite, tremolite, actinolite, and anthophyllite). Monogr. Eval. Carcinog. Risk Hum..

[2] Sayan, M. & Mossman, B. T. Erionite and asbestos in the pathogenesis of human malignant mesotheliomas. In: The molecular basis of human cancer (Coleman, W. B., Tsongalis, G. J. eds). Berlin, Springer, 287–295 (2017).

[3] Hawthorne FC (2012). Nomenclature of the amphibole supergroup. Am. Min..

[4] Wicks FJ, O’Hanley DS (1988). Serpentine minerals; structures and petrology. Rev. Min. Geochem..

[5] Gualtieri AF (1998). Crystal structure-crystal chemistry relationships in the zeolites erionite and offretite. Am. Min..

[6] Gualtieri, A. F., Bursi Gandolfi, N., Pollastri, S., Pollok, K. & Langenhorst, F. Where is iron in erionite? A multidisciplinary study on fibrous erionite-Na from Jersey (Nevada, USA). *Scientific reports* **6** (2016). 10.1038/srep37981 PMC5125093 27892512

[7] Donaldson K, Murphy FA, Duffin R, Poland CA (2010). Asbestos, carbon nanotubes and the pleural mesothelium: a review of the hypothesis regarding the role of long fiber retention in the parietal pleura, inflammation and mesothelioma. Part. Fiber Toxicol..

[8] Pollastri S (2014). The zeta potential of mineral fibers. J. Haz. Mat..

[9] Yarborough CM (2007). The risk of mesotelioma from exposure to chrysotile asbestos. Curr. Opin. In Pulm. Medicine.

[10] Oberdörster G (2000). Determinants of the pathogenicity of man-made vitreous fibers (MMVF). Int. Archives Occ. Env. Health.

[11] Bernstein DM, Chevalier J, Smith P (2005). Comparison of Calidria chrysotile asbestos to pure tremolite: final results of the inhalation biopersistence and histopathology examination following short-term exposure. Inhal. Toxicol..

[12] Utembe W, Potgieter K, Stefaniak AB, Gulumian M (2015). Dissolution and biodurability: Important parameters needed for risk assessment of nanomaterials. Particle Fiber Toxicol..

[13] McClellan RO, Hesterberg TW (1994). Role of Biopersistence in the Pathogenicity of Man-Made Fibers and Methods for Evaluating Biopersistence: A summary of Two Round-table Discussions. Env. Health Persp..

[14] Hesterberg TW (1998). Biopersistence of synthetic vitreous fibers and amosite asbestos in the rat lung following inhalation. Toxicol. App. Pharmacol..

[15] Hesterberg TW, Hart GA (2001). Synthetic vitreous fibers: A review of toxicology research and its impact on hazard classification. Critical Rev. in Tox..

[16] Jaurand MC, Bignon J, Sebastien P, Goni J (1977). Leaching of chrysotile asbestos in human lungs: Correlation with *in vitro* studies using rabbit alveolar macrophages. Environ. Res..

[17] Jaurand MC, Gaudichet A, Halpern S, Bignon J (1984). *In vitro* biodegradation of chrysotile fibers by alveolar macrophages and mesothelial cells in culture: comparison with a pH effect. *British*. J. Ind. Med..

[18] Nguea HD (2008). A new *in vitro* cellular system for the analysis of mineral fiber biopersistence. Arch. Toxicol..

[19] Lundborg M (1995). Phagolysosomal morphology and dissolution of cobalt oxide particles by human and rabbit alveolar macrophages. Exp. Lung Res..

[20] Potter RM, Mattson SM (1991). Glass fiber dissolution in a physiological saline solution. Glastechnische Berichte.

[21] Searl, A. & Buchanan, D. Measurement of the durability of man-made vitreous fibers. *Inst. Occ. Med. Research Report*, TM/00/03 (2000).

[22] Oze C, Solt KL (2010). Biodurability of chrysotile and tremolite asbestos in simulated lung and gastric fluids. Am. Min..

[23] Rozalen M, Ramos ME, Huertas FJ, Fiore S, Gervilla F (2013). Dissolution kinetics and biodurability of tremolite particles in mimicked lung fluids: Effect of citrate and oxalate. J. Asian Earth. Sci..

[24] Rozalen M (2014). Dissolution study of tremolite and anthophyllite: pH effect on the reaction kinetics. App. Geochemistry.

[25] Hume LA, Rimstidt JD (1992). The biodurability of chrysotile asbestos. Am. Min..

[26] Wagner JC, Skidmore JW (1965). Asbestos dust deposition and retention in rats. Ann. New York Academy Sci..

[27] Wagner JC, Berry G, Skidmore JW, Timbrell V (1974). The effects of the inhalation of asbestos in rats. British J. Cancer.

[28] Middleton AP, Beckett ST, Davis JM (1975). A study of the short-term retention and clearance of inhaled asbestos by rats, using UICC standard reference samples. Inhaled Particles.

[29] Bolton RE, Vincent JH, Jones AD, Addison J, Beckett ST (1983). An overload hypothesis for pulmonary clearance of UICC amosite fibres inhaled by rats. British J. Ind. Med..

[30] Churg A, Warnock ML (1980). Asbestos Fibers in the General Population 1–3. Am. Rev. Respiratory Disease.

[31] Langer, A. M. & Nolan, R. P. Fiber type and burden in parenchymal tissues of workers occupationally exposed to asbestos in the United States. In: Non-occupational Exposure to Mineral Fibers (Bignon, J., Peto, J. & Saracci, R. eds). Lyon, France, *IARC Scientific Publications* **90**, 330–335 (1989). 2545612

[32] Scholze H, Conradt R (1987). An *in vitro* study of the chemical durability of siliceous fibers. Ann. Occ. Hygiene.

[33] Muhle H, Bellmann B, Pott F (1994). Comparative investigations of the biodurability of mineral fibers in the rat lung. Env. Health Perspect..

[34] Searl A (1999). Biopersistence and durability of nine mineral fiber types in rat lungs over 12 months. Ann. Occ. Hygiene.

[35] Bernstein D (2013). Health risk of chrysotile revisited. Critical Rev. in Toxicol..

[36] Guldberg M (1998). Measurement of *in-vitro* fiber dissolution rate at acidic pH. *The Annals of Occ*. Hyg..

[37] Vigliaturo, R. Microstructures of potentially harmful fibrous minerals [PhD Dissertation]. Torino, University of Torino. Torino (Italy) (2015).

[38] Pollastri S (2016). Stability of mineral fibers in contact with human cell cultures. An *in situ* μXANES, μXRD and XRF iron mapping study. Chemosphere.

[39] Gualtieri AF (2017). New insights into the toxicity of mineral fibers: a combined *in situ* synchrotron micro-XRD and HR-TEM study of chrysotile, crocidolite, and erionite fibers found in the tissues of Sprague-Dawley rats. Toxicol. Lett..

[40] Chen Y, Brantley SL (1998). Diopside and anthophyllite dissolution at 25° and 90 °C and acid pH. Chem. Geol..

[41] Parry WT (1985). Calculated solubility of chrysotile asbestos in physiological systems. Env. Res..

[42] Schott J, Berner RA, Sjöberg EL (1981). Mechanism of pyroxene and amphibole weathering—I. Experimental studies of iron-free minerals. Geochim. Cosmochim. Acta.

[43] Delannay F, Cçekiewicz S (1985). Dark field TEM and XPS of proton exchanged erionite-offretite (T) zeolites. Zeolites.

[44] Yamamoto S (1996). Dissolution of zeolite in acidic and alkaline aqueous solutions as revealed by AFM imaging. J. Phys. Chem..

[45] Hartman RL, Fogler HS (2007). Understanding the dissolution of zeolites. Langmuir.

[46] Brantley, S. L. Kinetics of mineral dissolution. In: Kinetics of water-rock interaction (Brantley, S. L., Kubicki, J. D. & White, A. F. eds). New York, Springer 151–210 (2008).

[47] Hewkin DJ, Prince RH (1970). The mechanism of octahedral complex formation by labile metal ions. Coordination Chemistry Rev..

[48] Bernstein DM, Rogers R, Smith P (2004). The biopersistence of Brazilian chrysotile asbestos following inhalation. Inhal. Toxicol..

[49] Bernstein DM, Hoskins JA (2006). The health effects of chrysotile: current perspective based upon recent data. Regulatory Toxicol. Pharmacol..

[50] Speil S, Leineweber JP (1969). Asbestos minerals in modern technology. Environ. Res..

[51] Gaffney SH (2017). Anthophyllite asbestos: state of the science review. J. App. Toxicol..

[52] Aierken D (2014). Rat model demonstrates a high risk of tremolite but a low risk of anthophyllite for mesothelial carcinogenesis. Nagoya J. Med. Sci..

[53] Meurman LO, Pukkala E, Hakama M (1994). Incidence of cancer among anthophyllite asbestos miners in Finland. Occ. Env. Med..

[54] Wypych F, Adad LB, Mattoso N, Marangon AAS, Schreiner WH (2005). Synthesis and characterization of disordered layered silica obtained by selective leaching of octahedral sheets from chrysotile and phlogopite structures. J. Colloid. Interface Sci..

[55] Pollastri S (2015). The chemical environment of iron in mineral fibers. A combined X-ray absorption and Mössbauer spectroscopic study. J. Haz. Mat..

[56] De Meringo A, Morscheidt C, Thélohan S, Tiesler H (1994). *In vitro* assessment of biodurability: acellular systems. Environ. Health Perspect..

[57] Rietveld HM (1969). A profile refinement method for nuclear and magnetic structures. J. Appl. Cryst..

[58] Larson, A. C. & Von Dreele, R. B. GSAS. Generalized Structure Analysis System. Los Alamos Nat. Lab., New Mexico, LAUR (1994).

[59] Toby BH (2001). EXPGUI, a graphical user interface for GSAS. J. App. Cryst..

[60] Gualtieri AF (2000). Accuracy of XRPD QPA using the combined Rietveld–RIR method. J. App. Cryst..

[61] Brantley, S. L. & Conrad, C. F. Analysis of rates of geochemical reactions. In: Kinetics of water-rock interaction (Brantley, S. L., Kubicki, J. D. & White, A. F. eds). New York, Springer 1–37 (2008).

