# Supplementary material for: In vitro acellular dissolution of mineral fibres: A comparative study
Source: Sci Rep. 2018 May 4;8:7071. doi: 10.1038/s41598-018-25531-4 (PMC5935704; doi:10.1038/s41598-018-25531-4)
Supplement: Supplementary file 1 — Supplementary Material [file 41598_2018_25531_MOESM1_ESM.pdf]

In vitro acellular dissolution of mineral fibres.

A comparative study

Alessandro F. Gualtieri[1*], Simone Pollastri[1], Nicola Bursi Gandolfi[1], and Magdalena Lassinantti Gualtieri[2]

[1]Department of Chemical and Geological Sciences, The University of Modena and Reggio Emilia, Modena, Italy; [2]Department of Engineering "Enzo Ferrari", The University of Modena and Reggio Emilia, Modena, Italy

SUPPLEMENTARY MATERIAL

Results of the Rietveld refinements

UICC standard amosite*

Time (h)	amosite (wt%)	amorphous (wt%)	R_{wp}**(%)	R_p**(%)
0	100	0	10.2	8.1
24	0.99(2)	0.01(2)	10.1	8.2
48	0.99(2)	0.01(2)	10.4	8.3
168	0.98(1)	0.02(1)	10.3	8.4
336	0.98(1)	0.02(1)	10.2	8.1
720	0.98(2)	0.02(2)	9.7	7.6
1440	0.97(2)	0.03(2)	9.6	7.4
2160	0.97(2)	0.03(2)	9.5	7.5
3600	0.96(2)	0.04(2)	9.4	7.6
5040	0.96(2)	0.04(2)	10.2	8.0
6480	0.96(2)	0.04(2)	10.2	7.9

*impurities: calcite, hematite and quartz (<1wt%) not detected with XRPD but observed with SEM/EDS; **as defined in GSAS (Larson and Von Dreele, 1994).

UICC standard anthophyllite asbestos*

Time (h)	anthophyllite (wt%)	biotite (wt%)	clinochlore (wt%)	talc (wt%)	amorphous (wt%)	R_{wp}** (%)	R_p** (%)
0	89.2(2)	1.4(4)	1.7(3)	7.7(3)	0	6.15	4.31
24	89.2(3)	1.4(4)	1.7(3)	7.7(3)	0	6.13	4.31
48	90.3(2)	1.4(4)	1.3(3)	7.0(3)	0	6.37	4.35
168	92.5(2)	1.3(4)	1.1(3)	5.1(3)	0	6.26	4.29
336	94.2(3)	1.3(4)	1.0(3)	2.5(3)	1.0(1.3)	6.12	4.20
720	96.1(2)	1.2(4)	0.5(3)	1.0(3)	1.2(1.2)	6.11	4.22

1440	97.6(2)	1.1(4)	0	0	1.3(6)	6.64	4.51
2160	97.3(2)	1.1(4)	0	0	1.6(6)	6.56	4.40
3600	96.9(2)	1.1(4)	0	0	2.0(6)	6.14	4.30
5040	97.0(3)	1.0(4)	0	0	2.0(7)	6.88	4.45
6480	96.9(3)	1.0(4)	0	0	2.1(7)	6.68	4.90

*impurities: biotite 1.4(2) wt%, clinochlore/vermiculite 1.7(3) wt% and talc 7.7(4) wt%; **as defined in GSAS (Larson and Von Dreele, 1994).

UICC standard crocidolite*

Time (h)	crocidolite (wt%)	amorphous (wt%)	R_{wp}**(%)	R_p**(%)
0	100	0	12.5	9.4
24	100	0	12.2	9.2
48	100	0	12.0	9.1
168	0.99(2)	0.01(2)	12.4	9.4
336	0.99(2)	0.01(2)	11.8	8.9
720	0.985(2)	0.015(2)	11.9	9.0
1440	0.98(2)	0.02(2)	12.6	9.4
2160	0.98(2)	0.02(2)	12.7	9.5
3600	0.97(2)	0.03(2)	12.7	9.6
5040	0.97(2)	0.03(2)	13.0	9.8
6480	0.965(2)	0.035(2)	12.4	9.3

*impurities: hematite, magnetite, and quartz (<1wt%) not detected with XRPD but observed with SEM/EDS; **as defined in GSAS (Larson and Von Dreele, 1994).

Tremolite asbestos from Val d'Ala, Turin (Italy)*

Time (h)	tremolite (wt%)	antigorite (wt%)	clinochlore (wt%)	amorphous (wt%)	R_{wp}** (%)	R_p** (%)

0	92.5(3)	5.1(5)	2.4(3)	0	15.88	10.71
24	95.2(2)	2.5(4)	2.3(3)	0	15.80	10.70
48	95.9(2)	2.0(4)	2.1(3)	0	15.76	10.64
168	96.0(2)	1.5(4)	1.5(3)	1.0(1.0)	15.81	10.66
336	96.6(2)	1.2(5)	1.0(3)	1.2(1.0)	16.54	11.23
720	97.0(2)	1.0(4)	0.7(3)	1.3(9)	16.57	11.20
1440	98.2(2)	0	0	1.8(2)	18.01	11.44
2160	98.2(2)	0	0	1.8(2)	17.21	10.53
3600	98.1(2)	0	0	1.9(2)	18.30	10.62
5040	98.0(2)	0	0	2.0(2)	17.02	10.98
6480	98.0(2)	0	0	2.0(2)	17.10	10.83

*impurities: antigorite and clinochlore; **as defined in GSAS (Larson and Von Dreele, 1994).

Balangero (Torino, Italy) chrysotile*

Time (h)	chrysotile (wt%)	amorphous (wt%)	R_{wp}**(%)	R_p**(%)
0	98.0(5)	2.0(5)	13.75	9.09
4	92.8(5)	2.2(5)	13.45	9.80
8	86.3(5)	13.7(5)	13.44	9.90
24	80.1(5)	19.9(5)	13.56	9.04
48	73.8(5)	26.2(5)	12.44	8.21
168	56.2(5)	43.8(5)	12.65	7.33
336	33.2(5)	66.8(5)	11.43	6.76
720	25.2(5)	74.8(5)	10.88	7.28
1440	20.2(6)	79.8(6)	9.84	6.99
2160	13.3(6)	86.7(6)	9.65	6.86
3600	0	100	9.54	6.87

*impurities: antigorite, balangeroite, calcite, clinochlore, diopside, dolomite, magnetite, microcline, plagioclase, and talc. All the phases are <1wt% , observed with SEM/EDS but not included in the refinements; **as defined in GSAS (Larson and Von Dreele, 1994).

UICC standard chrysotile "B" asbestos*

Time (h)	chrysotile (wt%)	amorphous (wt%)	R_{wp}**(%)	R_p**(%)
0	100	0	6.87	5.24
4	92.8(4)	7.2(4)	6.58	5.02
8	85.3(4)	14.7(4)	8.78	6.56
24	78.1(4)	21.9(4)	5.86	4.41
48	66.8(4)	33.2(4)	6.27	4.31
168	50.2(4)	49.8(4)	6.70	4.05
336	26.1(4)	73.9(4)	13.83	6.29
720	15.0(4)	85.0(4)	13.76	6.20
1440	5.2(4)	94.8(4)	5.07	3.80
2160	0	100	5.14	3.87
3600	0	100	5.32	3.98

*impurities: brucite, calcite, clinochlore, dolomite, magnetite, microcline, pyroaurite and talc. All the phases are <1wt% , observed with SEM/EDS but not included in the refinements; **as defined in GSAS (Larson and Von Dreele, 1994).

Valmalenco (Sondrio, Italy) chrysotile*

Time (h)	chrysotile (wt%)	amorphous (wt%)	R_{wp}**(%)	R_p**(%)
0	100	0	12.76	9.94
4	93.8(5)	6.2(5)	13.35	9.58
8	86.3(5)	13.7(5)	11.96	9.13

24	80.1(5)	19.9(5)	7.27	5.43
48	67.8(5)	32.2(5)	15.45	10.61
168	53.2(5)	46.8(5)	11.62	5.99
336	29.1(5)	70.9(5)	10.22	5.78
720	21.0(5)	79.0(5)	9.32	7.05
1440	9.2(5)	90.8(5)	7.45	5.21
2160	0	100	5.55	4.01
3600	0	100	5.64	4.12

*impurities: calcite, forsterite, magnetite, quartz, lizardite/antigorite, clinochlore. All the phases are <1wt% , observed with SEM/EDS but not included in the refinements; **as defined in GSAS (Larson and Von Dreele, 1994).

Erionite-Na from Jersey, Nevada (USA)*

Time (h)	erionite (wt%)	Amorphous (wt%)	R_{wp}**(%)	R_p** (%)
0	100	0	11.45	8.05
24	100	0	11.56	8.07
48	99.7(2)	0.3(2)	15.73	10.29
168	99.1(2)	0.9(2)	13.87	9.43
336	99.0(2)	1.0(2)	12.97	9.41
720	99.3(2)	0.7(2)	15.07	9.83
1440	99.1(2)	0.9(2)	16.11	10.92
2160	98.5(2)	1.5(2)	16.15	10.98
3600	98.1(2)	1.9(2)	13.53	9.28
5040	98.2(2)	1.8(2)	13.52	9.51
6480	98.1(2)	1.9(2)	13.67	9.35

*impurities: clinoptilolite (<1wt%) not included in the refinement; **as defined in GSAS (Larson and Von Dreele, 1994).

Determination of the kinetic parameters

The actual mass of the mineral fibers dissolved with time, accurately determined from the quantitative phase analysis, was converted into moles (fiber mass/fiber molar weight M) to obtain a plot of moles vs. time (s) like the molal concentration vs. time plots shown in Hume and Rimstidt (Fig. 1 of the manuscript)[25]. The fit of the final part of the curve was accomplished with a first order equation that yielded the apparent rate constant k (s^{-1}). The calculated value of k was used to determine the apparent dissolution rate R ($mol \cdot g^{-1} s^{-1}$) from eq. (1) valid for batch reactors[61]:

$$R = \frac{k}{SSA \cdot m} \quad (1)$$

with SSA=measured initial specific surface area (m^2/g) (Table 1 of the manuscript) and m=initial mass of the sample (g). SSA determined with the BET method is assumed to be an acceptable approximation of the reactive surface area of the non-porous samples (amphibole and chrysotile asbestos) whereas the external surface area determined with the t-plot method is assumed to be a better approximation of the reactive surface area for the zeolite erionite (see experimental section).

In the case of amphibole fibers and erionite, to which eq. (1) was applied, the dissolution rates are very slow and initial SSA is assumed to be constant during the experiments. This approximation is acceptable if we consider that the amount of dissolved fibers in the time span of the experiments is limited. For such fibers, the initial measured SSA_{BET} is used as input value in the equation for amphibole asbestos species whereas external surface area is used in the erionite case. This assumption does not apply to chrysotile asbestos fibers which completely dissolve during the time span of the experiments. For chrysotiles, a geometric model was developed, assuming surface-advancing reaction and samples composed of equally sized fibers. Our model explicitly considers fiber facets and lengths with equal dissolution rates. This should be considered an approximation as different crystal facets may not dissolve at the same rate.

The SSA (m^2/g) and true density (ρ) of each chrysotile mineral were used as input parameters to calculate; i) the initial fiber radius (r_0); ii) the corrosion distance at time t (X_t) and thus the radius at time t ($r_t=r_0-X_t$); iii) the surface area at time t (A_t). The model is based on the following expressions relating the surface area (A_f), volume (V_f) and mass (m_f) of a fiber (approximated to be a perfect cylinder) having radius r, length L and density ρ;

$$A_f = 2r\pi L + 2r^2\pi \quad (2)$$

$$V_f = r^2 \pi L \quad (3)$$

$$m_f = V_f \rho \quad (4)$$

Now, *SSA* of a population composed of equally sized fibers can simply be expressed as the number of fibers in one gram (n) multiplied by A_f;

$$SSA = A_f n \quad (5)$$

where:

$$n = \frac{1}{m_f} \quad (6)$$

Combining the above eq. (5) and (6), the following relationship between r_f and L_f is obtained:

$$r_f = \frac{2L_f}{SSA \cdot L_f \cdot \rho - 2} \quad (7)$$

Eq. (7) was used to calculate r_0, using known values of SSA, ρ, and fiber length (L_0). This last parameter does not significantly influence r_0, as clear from eq. (7). Now, the weight fraction of undissolved material at time *t*, a parameter known experimentally, can be expressed as:

$$\frac{m_t}{m_0} = \frac{n \cdot V_{f\,t} \rho}{n \cdot V_{f_0} \rho} = \frac{r_t^{\,2} \pi L_t}{r_0^{\,2} \pi L_0} \quad (8)$$

where $r_t = r_0 - x_t$, $L_t = L_0 - 2x_t$ and x_t is the corrosion distance at time *t.*

This cubic equation was resolved with respect to X_t, thus allowing to calculate r_t and L_t. The area fraction of undissolved material at time t can be thus calculated, in combination with the above equations (2-8), by eq. (9).

$$\frac{A_t}{A_0} = \frac{A_{ft} n}{SSA} \quad (9)$$

The variation of A (m^2) with time is then used to determine the apparent rate constant k from eq. (10)[36]:

$$\frac{dm}{dt} = -k \cdot A(t) \quad (10)$$

In this way, the rate constant also includes the contribution of the variation of surface area with time. k is then used to determine the apparent dissolution rate R from eq. (1)[61]. The fit for the three chrysotile sample is shown in Fig. 1 of the manuscript.

To verify if the kinetic curves of dissolution determined from our gravimetry experiments are accurate, comparison with experimental data obtained from the classical analysis of ions (Si, Fe, Mg) released in suspension was accomplished for a chrysotile sample. The sample selected for this cross-test was the Balangero chrysotile. The kinetics of dissolution was monitored in a time span of three months by measuring the amount (in ppb) of released Si, Fe, and Mg ions using inductively coupled plasma optical emission spectrometry (ICP-OES). The figure below reports the normalized data for the Balangero chrysotile. The mass data are the same values reported in Fig. 2(b) of the manuscript whereas Mg, Si and Fe are normalized concentrations (in ppb). A good match is observed for the different data sets witnessing the accuracy of the kinetic data determined from our gravimetry experiments.

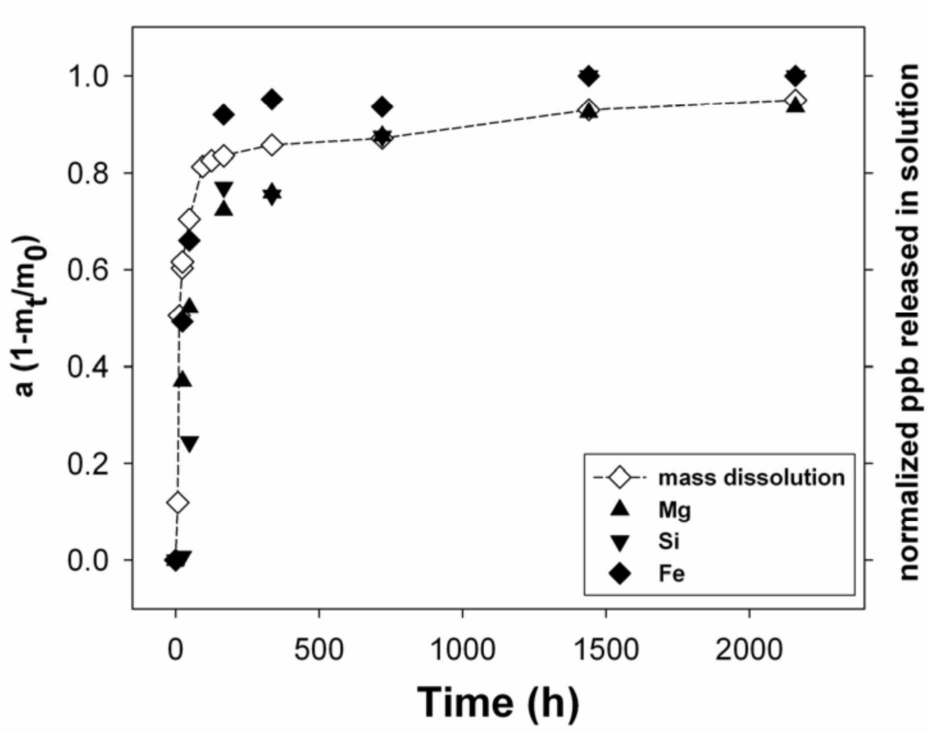

For all the fibers, the estimated lifetime t of a fiber was determined using eq. (11)[23,25]:

$$t = \frac{3d}{4VR} \quad (11)$$

where d=fiber diameter (m): a diameter of 0.25 µm (0.25×10^{-6} m) was assumed for the calculation; V= molar volume ($m^3\cdot mol^{-1}$), R= apparent dissolution rate normalized to the surface area A (see above).

Results

Dissolution curves for chrysotile and fibrous amphibole. The dissolution of the UICC sample is faster than the others with the Balangero chrysotile exhibiting the slowest dissolution rate. The dissolution of erionite is faster than the amphibole species. Tremolite and anthophyllite asbestos dissolve faster than the iron rich species crocidolite and amosite with amosite showing the slowest dissolution rate.

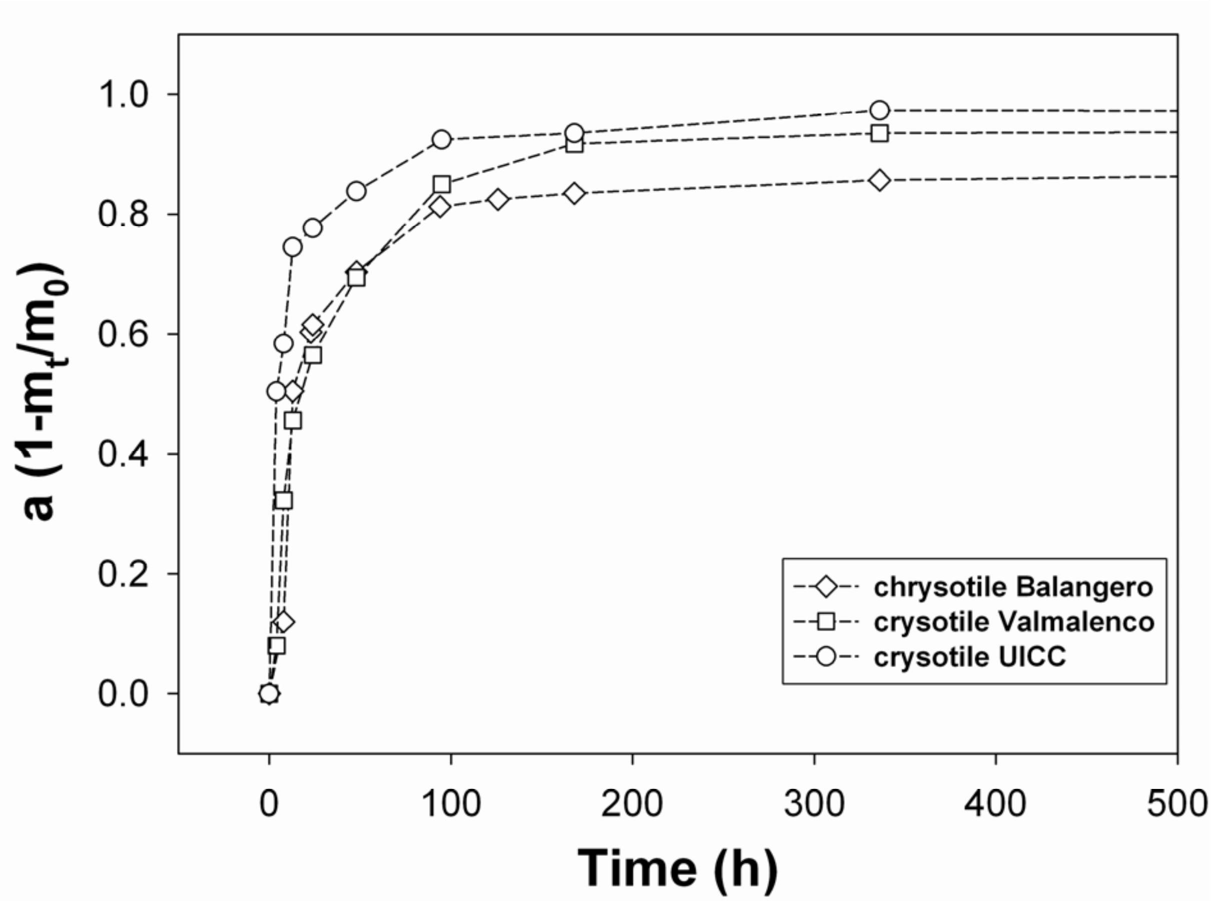

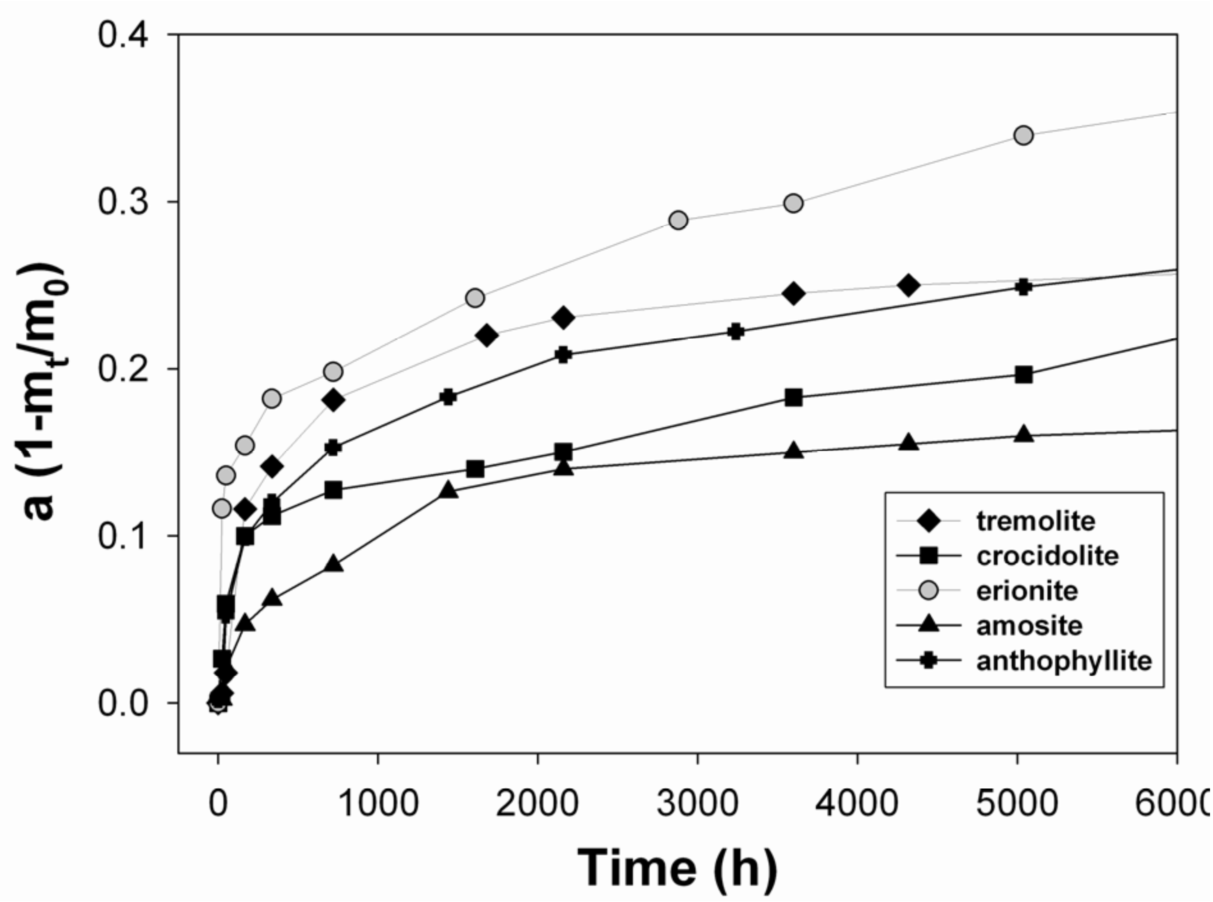

References (from the body of the manuscript)

23. Rozalen, M., Ramos, M.E., Huertas, F.J., Fiore, S. & Gervilla, F. Dissolution kinetics and biodurability of tremolite particles in mimicked lung fluids: Effect of citrate and oxalate. *J. Asian Earth. Sci*. **77**, 318-326 (2013).

25. Hume, L.A. & Rimstidt, J.D. The biodurability of chrysotile asbestos. *Am. Min*. **77**, 1125-1128 (1992).

36. Guldberg, M., Christensen, V.R., Perander, M., Zoitos, B., Koenig, A.R. *et al.* Measurement of in-vitro fiber dissolution rate at acidic pH. *The Annals of Occ. Hyg*. **42**, 233-243 (1998).

61. Brantley, S.L. & Conrad, C.F. Analysis of rates of geochemical reactions. In: Kinetics of water-rock interaction (Brantley, S.L., Kubicki, J.D., White, A.F., eds). New York, Springer 1-37 (2008).
